# Corresponding Mitochondrial DNA and Niche Divergence for Crested Newt Candidate Species

**DOI:** 10.1371/journal.pone.0046671

**Published:** 2012-09-28

**Authors:** Ben Wielstra, Wouter Beukema, Jan W. Arntzen, Andrew K. Skidmore, Albertus G. Toxopeus, Niels Raes

**Affiliations:** 1 Naturalis Biodiversity Center, Leiden, The Netherlands; 2 University of Twente, Faculty of Geo-Information Science and Earth Observation – ITC, Enschede, The Netherlands; Onderstepoort Veterinary Institute, South Africa

## Abstract

Genetic divergence of mitochondrial DNA does not necessarily correspond to reproductive isolation. However, if mitochondrial DNA lineages occupy separate segments of environmental space, this supports the notion of their evolutionary independence. We explore niche differentiation among three candidate species of crested newt (characterized by distinct mitochondrial DNA lineages) and interpret the results in the light of differences observed for recognized crested newt species. We quantify niche differences among all crested newt (candidate) species and test hypotheses regarding niche evolution, employing two ordination techniques (PCA-env and ENFA). Niche equivalency is rejected: all (candidate) species are found to occupy significantly different segments of environmental space. Furthermore, niche overlap values for the three candidate species are not significantly higher than those for the recognized species. As the three candidate crested newt species are, not only in terms of mitochondrial DNA genetic divergence, but also ecologically speaking, as diverged as the recognized crested newt species, our findings are in line with the hypothesis that they represent cryptic species. We address potential pitfalls of our methodology.

## Introduction

Phylogeography has yielded a wealth of information by documenting geographical genetic variation [1]. One key finding is the frequent presence of extensive mitochondrial DNA variation within taxa, not matched by morphological differentiation [2]. Simply translating such ‘cryptic diversity’ to species status would explicitly interpret mitochondrial DNA divergence as reflecting evolutionary independence. However, genetic divergence of mitochondrial DNA is not necessarily a suitable proxy for the presence of species boundaries [3], [4]. Ecological divergence can act as a barrier to gene flow and thus promote reproductive isolation and speciation [5]–[9]. If geographical populations characterized by distinct mitochondrial DNA lineages (hereafter referred to as ‘candidate species’) also occur under different ecological conditions, this increases support for their treatment as distinct species.

**Figure 1:**
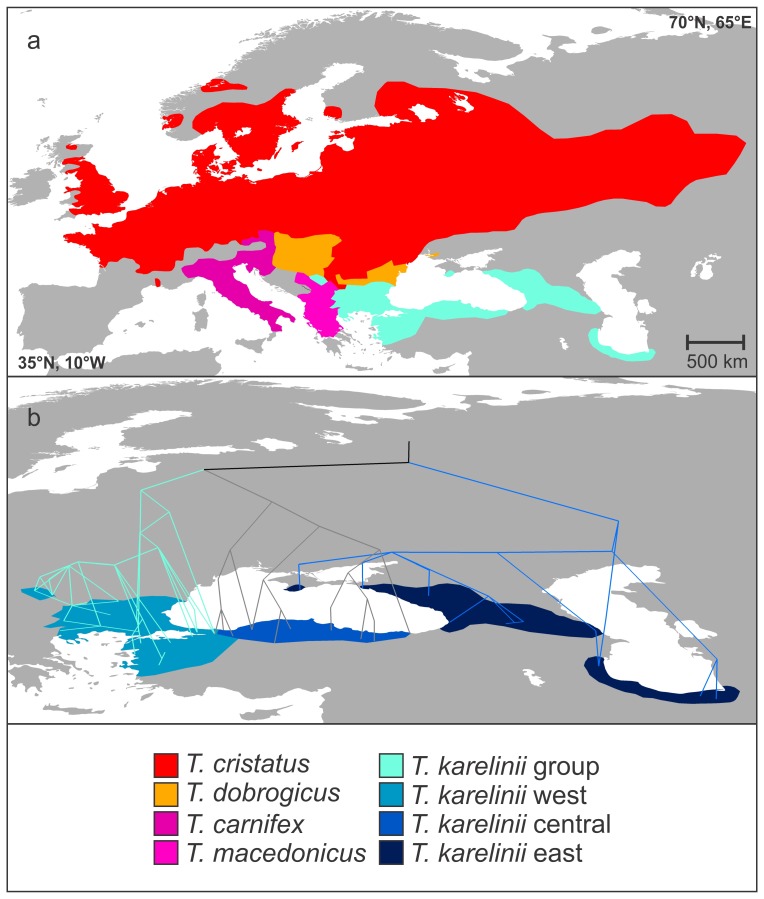
The distribution of the crested newt *Triturus cristatus* superspecies. Fig. 1a shows the distribution of the four recognized crested newt species and the *Triturus karelinii* group. Fig. 1b shows the distribution of the distinct eastern, central and western mitochondrial DNA lineages comprising the *Triturus karelinii* group (sensu [11]), with a geophylogeny superimposed (created with GeoPhyloBuilder [31]).

The crested newt *Triturus cristatus* superspecies consists of five parapatric groups (Fig. 1a). Four of these, *T. carnifex*, *T. macedonicus*, *T. cristatus* and *T. dobrogicus*, are recognized as distinct species [10]. The systematics of the fifth group, traditionally referred to as *T. karelinii*, is more complex. In a previous phylogeographic study [11], we uncovered three geographically structured mitochondrial DNA lineages in the *T. karelinii* group (Fig. 1b). These lineages show a level of genetic divergence comparable to that among the recognized crested newt species, with pairwise distances of full mitogenomic sequences in the range of 0.041–0.058 versus 0.039–0.064 [12]. The three mitochondrial DNA lineages constituting the *T. karelinii* group are hereafter referred to as candidate species. Divergence times among the recognized and candidate crested newt species are estimated to be of Late Miocene (c.11.6-5.3Ma) origin. The recognized species are morphologically distinct [13] and represent discrete nuclear gene pools ([10] J.W. Arntzen and collaborators, submitted). There are currently no morphological grounds for distinguishing the three *T. karelinii* candidate species (body build and skull shape [13], [14]) and information on nuclear gene flow is as yet lacking. This raises the question whether the three mitochondrial DNA lineages comprising the *T. karelinii* group represent a single or several species.

We explore the use of niche divergence as a criterion for assigning species status to candidate species identified with mitochondrial DNA. To this aim, we use a statistical framework recently proposed by Broennimann and collegues [15], which quantifies niche differences and tests hypotheses regarding niche evolution. We determine niche differences among the three crested newt candidate species and interpret these differences by treating niche divergence among recognized species of crested newt as a benchmark, meaning we treat the degree of niche divergence between recognized congeneric species as a minimum threshold that candidate species should express to qualify for species status. We suggest that the similar niche differentiation among candidate species as among recognized species that we identify is in support of species status for the three candidate crested newt species. However, we do highlight considerations regarding the application and interpretation of niche differentiation in systematics.

## Methods

### Distribution and environmental data

We composed a dataset of 2404 crested newt occurrences covering all recognized species and partitioned those for the *T. karelinii* group into three classes according to mitochondrial DNA: 120 *T. carnifex*, 1698 *T. cristatus*, 136 *T. dobrogicus*, 139 *T. macedonicus*, 135 *T. karelinii* west, 32 *T. karelinii* central and 144 *T. karelinii* east (see Dataset S1). For ecological data layers, we used bioclimatic variables at 2.5 arcminute resolution (c. 5×5 km) available from the WorldClim database 1.4 ([16] http://www.worldclim.org). It is recommended to focus on data layers that are deemed biologically meaningful based on life history knowledge of the model system [17], [18]. Considering that the recognized crested newt species differ in their requirements concerning the availability of water bodies for breeding [13], we selected a set of layers that encompasses seasonal variation in evaporation and precipitation, in casu bio10  =  mean temperature of warmest quarter, bio11  =  mean temperature of coldest quarter, bio15  =  precipitation seasonality, bio16  =  precipitation of wettest quarter, and bio17  =  precipitation of driest quarter. To set our study area we drew a buffer around all crested newt localities [19], using a 200 km radius following VanDerWal et al. [20].

### Quantifying niche overlap

To calibrate niches and measure niche overlap, we applied a recently presented framework by Broennimann *et al*. [15] implemented in R [21]. We used the two best performing niche calibration techniques: Principal Component Analysis (PCA) calibrated on the entire environmental space of the study area – PCA-env [15] and Ecological Niche Factor Analysis – ENFA [22]. In PCA-env, a PCA is conducted to transform the climate layers into a reduced number of linearly uncorrelated variables, i.e. principal components [15]. The first component accounts for as much of the variability in the original variables as possible and each following component accounts for as much of the remaining variability. In PCA-env the PCA is calibrated on the entire study areas(including species occurrences). Differences in the position of (candidate) species along the principal components reflect their environmental differences. ENFA similarly is an ordination technique. It compares the environmental conditions experienced by a (candidate) species' distribution to that present in the entire study area [15], [22]. The principal components in ENFA have a direct ecological interpretation. The first component reflects the marginality: the ecological distance between the (candidate) species' optimum and the mean conditions of the study area. The second component reflects specialization: the ratio of the ecological variance in the study area to that observed for the (candidate) species.

Application of the framework firstly encompasses the calibration of the niche and the occurrence density. Environmental space is defined by the first two axes in which the chosen ordination technique summarizes the environmental conditions in the study area. This environmental space is divided into a grid of *r* x *r* cells (we use r = 100 as in [15]) bounded by the minimum and maximum values occurring in the study area. Each cell in environmental space represents a unique combination of environmental conditions present in one or more of the grid cells in geographical space. To correct for potential sampling bias, a smoothed occurrence density for each (candidate) species in each grid cell is estimated using a kernel density function [15]. Subsequently, niche overlap for pairs of (candidate) species is calculated using Schoener's *D* [23]. This metric ranges from 0, which means niches are completely dissimilar, to 1, which signifies that niches completely overlap [15].

### Interpreting niche differentiation

To test whether pairwise niche overlap values for candidate species are significantly more similar than those for recognized species, we conducted a *t*-test using IBM SPSS 20.

The Broennimann *et al*. framework tests two hypotheses regarding niche evolution (as in [24]). The niche equivalency test explores whether niches of two (candidate) species are identical. The occurrences for a pair of (candidate) species are pooled, two random sets of occurrences with the same original sample sizes are extracted, and the overlap scores are determined. By repeating this procedure (here a hundred times), a null distribution of overlap scores is obtained, to which the actual overlap score for the two (candidate) species is compared. If the actual niche overlap is significantly smaller, this means the (candidate) species occupy distinct segments of environmental space.

The niche similarity test explores whether the niche overlap between (candidate) species is larger than expected by chance, based on the different environmental conditions they encounter. We defined this ‘background’ for each (candidate) species by drawing a 200 km buffer around its occurrences. The niche similarity test determines niche overlap of one (candidate) species with a random model based on the background of the other (using as much random samples as there are occurrences for the other), and the reciprocal. This is repeated (here a hundred times), resulting in two null distributions, one for each (candidate) species, to which the actual niche overlap between the two (candidate) species is subsequently compared. A statistically significantly higher niche overlap value indicates that the niches of the two (candidate) species are more similar than expected by chance.

## Results

**Figure 2:**
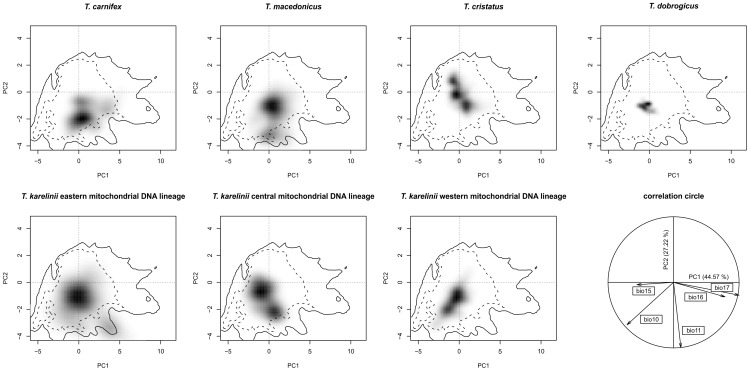
Niches of the different crested newt (candidate) species based on PCA-env. Each (candidate) species' niche is displayed on the same referential: a multi-dimensional scale represented by the first two axes of a principal component analyses summarizing the entire study area. Grey shading reflects the density of the occurrences of each (candidate) species in each cell. The solid and dashes contour lines illustrate 100% and 50% of the available environment in the study area. The correlation circle (bottom left) shows the contribution of the climatic variables on the two axes of the PCA and the percentage of inertia explained by the two axes.

The environmental space occupied by each (candidate) species as determined by PCA-env is shown in Fig. 2 and results from ENFA can be found in Figure S1. Both the recognized and the candidate species differ in their position in environmental space. Indeed, the niche equivalency tests showed that niche overlap among (candidate) species is significantly smaller than the null distribution (*p* = 0.198), meaning they occupy non-identical niche space. Niche overlap values calculated with PCA-env and ENFA can be found in table 1. The *t*-test shows that niche overlap among the three candidate species is not significantly larger from that among the recognized species, based on both PCA-env (*p* = 0.338) and ENFA (*p* = 0.165). Part of the similarity tests are significant (Table 1), meaning that in these cases newts' niches are more similar than their backgrounds are; this is relatively more often the case for candidate species (4 and 3 out of 6 tests based on PCA-env and ENFA) than for recognized species (5 and 2 out of 12 tests).

**Table 1:** Niche overlap values and results of similarity tests for each pair of crested newt candidate species (the three *Triturus karelinii*'s) and recognized species as determined with PCA-env (below diagonal) and ENFA (above diagonal).

	*T. karelinii* east	*T. karelinii* central	*T. karelinii* west	*T. carnifex*	*T. macedonicus*	*T. cristatus*	*T. dobrogicus*
*T. karelinii* east	-	0.244^ns/*^	0.559	0.366^*/ns^	0.456^ns/*^	0.347	0.060
*T. karelinii* central	0.329^*/*^	-	0.422^*/*^	0.345	0.450	0.254	0.252
*T. karelinii* west	0.209	0.281^*/*^	-	0.325	0.411^*/*^	0.399	0.318
*T. carnifex*	0.408^*/*^	0.498^ns/*^	0.239^*/ns^	-	0.309	0.446	0.182^*/*^
*T. macedonicus*	0.485^*/*^	0.391^ns/*^	0.162	0.448^ns/*^	-	0.319	0.135
*T. cristatus*	0.128^*/*^	0.124^ns/*^	0.182	0.253^*/ns^	0.120^*/*^	-	0.224
*T. dobrogicus*	0.041	0.090	0.188	0.055^*/ns^	0.040	0.142	-

Similarity tests compare one (candidate) species with the background of the other and the reverse. If one or both comparisons are significant, results for the comparison of (candidate) species listed from top to bottom with the background of the one listed from left to right are noted before the slash; after the slash the reverse combination is noted. *  =  significantly larger (p<0.05) and ns  =  non-significant.

## Discussion

The three candidate species comprising the *T. karelinii* group are, not only in terms of mitochondrial DNA genetic divergence, but also ecologically speaking as diverged as the recognized crested newt species: niche overlap among them is not significantly higher than that among recognized crested newt species. The niche equivalency tests reveal that all (candidate) species occupy different climatic corners of environmental space (Fig. 2, Figure S1). Although the niche overlap values among candidate species are not significantly higher than those among recognized species, the niche similarity tests show that candidate species relatively more often show a higher degree of overlap than would be expected based on the differentiation of their ecological backgrounds. This would suggest the candidate species occupy relatively diverged backgrounds.

The detailed knowledge regarding morphological and genetic differentiation that has become available for other crested newts led to their recognition as distinct species ([13], [25], [26] J.W. Arntzen and collaborators, submitted). Such information is as yet inconclusive (morphology) or lacking (nuclear genome) for the *T. karelinii* group. However, next to the previously found mitochondrial DNA divergence among candidate species comparable to that among recognized crested newt species, there is now another line of evidence suggesting the presence of three morphologically cryptic species: the amount of niche divergence among candidate species is similar to that among recognized crested newt species too. To test the prediction of explicit barriers to nuclear gene flow among the three candidate species comprising the *T. karelinii* group, a multimarker nuclear DNA phylogeography is required.

Care should be taken when interpreting niche divergence in systematics. The environmental conditions where candidate species are observed to occur should not be confused with those where they could occur [27]. The potential niche envelopes those environmental conditions that exists in nature, under which a candidate species could persist and reproduce. The realized niche comprises the portion of the potential niche that is actually occupied, given that historical and biotic factors pose further restriction on a candidate species' distribution [27]. It follows that although candidate species may differ in their realized niche, they might still possess the same potential niche. Differences among candidate species' realized niches could reflect niche evolution, in other words, an actual change in the potential niche (and a mechanism resulting in ecological isolation). However, these differences could also reflect niche plasticity, with candidate species possessing the same potential niche despite expressing a different realized niche. Controlled field experiments are required to distinguish niche plasticity from niche evolution.

The integration of spatial ecological methods in mitochondrial DNA phylogeographical surveys provides a powerful tool, aiding the delineation of cryptic species [28], [29]. Using phylogenetically informed partitioning of occurrence data is facilitated by the rapid increase in the availability of both sequence data (especially via mitochondrial DNA barcoding initiatives) and occurrence data [30]. The approach outlined in this paper will aid systematicists and conservationists alike in assessing the potential species status of distinct mitochondrial DNA lineages, in relatively little time and at low cost. However, we urge care to be taken not to over interpret indicative results, but prioritize such cases in further research.

## Supporting Information

Dataset S1 **Occurrence data for crested newt (candidate) species.**
(XLS)

Figure S1 **ENFA results for each pairwise comparison of (candidate) species.**
(PDF)
