# Supplementary material for: Corresponding Mitochondrial DNA and Niche Divergence for Crested Newt Candidate Species
Source: PLoS One. 2012 Sep 28;7(9):e46671. doi: 10.1371/journal.pone.0046671 (PMC3460878; doi:10.1371/journal.pone.0046671)
Supplement: Figure S1 — ENFA results for each pairwise comparison of (candidate) species. (PDF) [file pone.0046671.s002.pdf]

Figure S1. The results of the ENFA analysis for each pairwise comparison of (candidate) species. The x-axis shows marginality and the y-axis specialization. Grey shading shows the density of the occurrences of each (candidate) species' by cell. The solid and dashes contour lines illustrate 100% and 50% of the available environment in the study area. The environmental background looks different for each pairwise comparison because it is based on randomly drawn pseudo-absence data. The correlation circles (right) show the contribution of the climatic variables to the two axes.

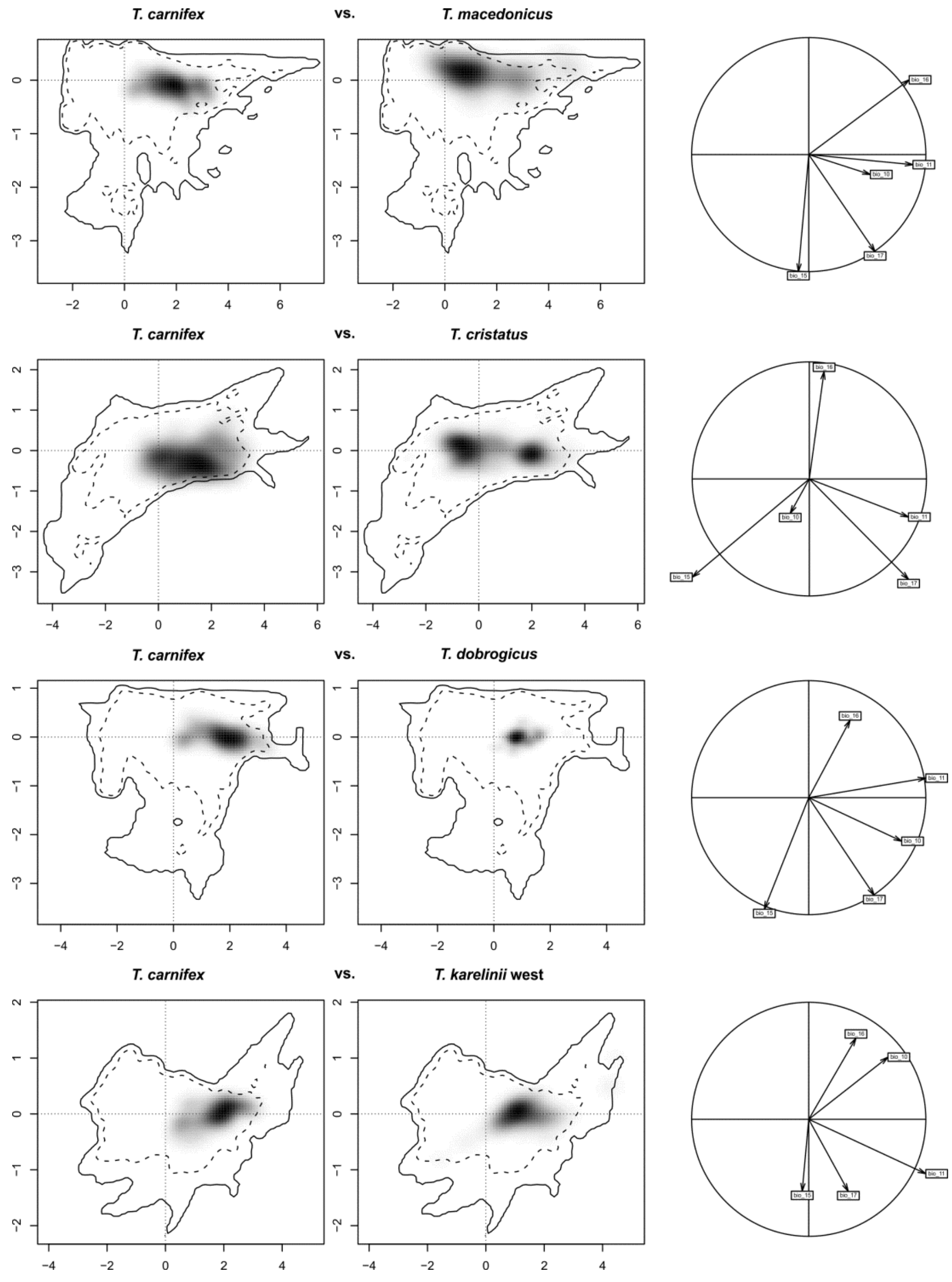

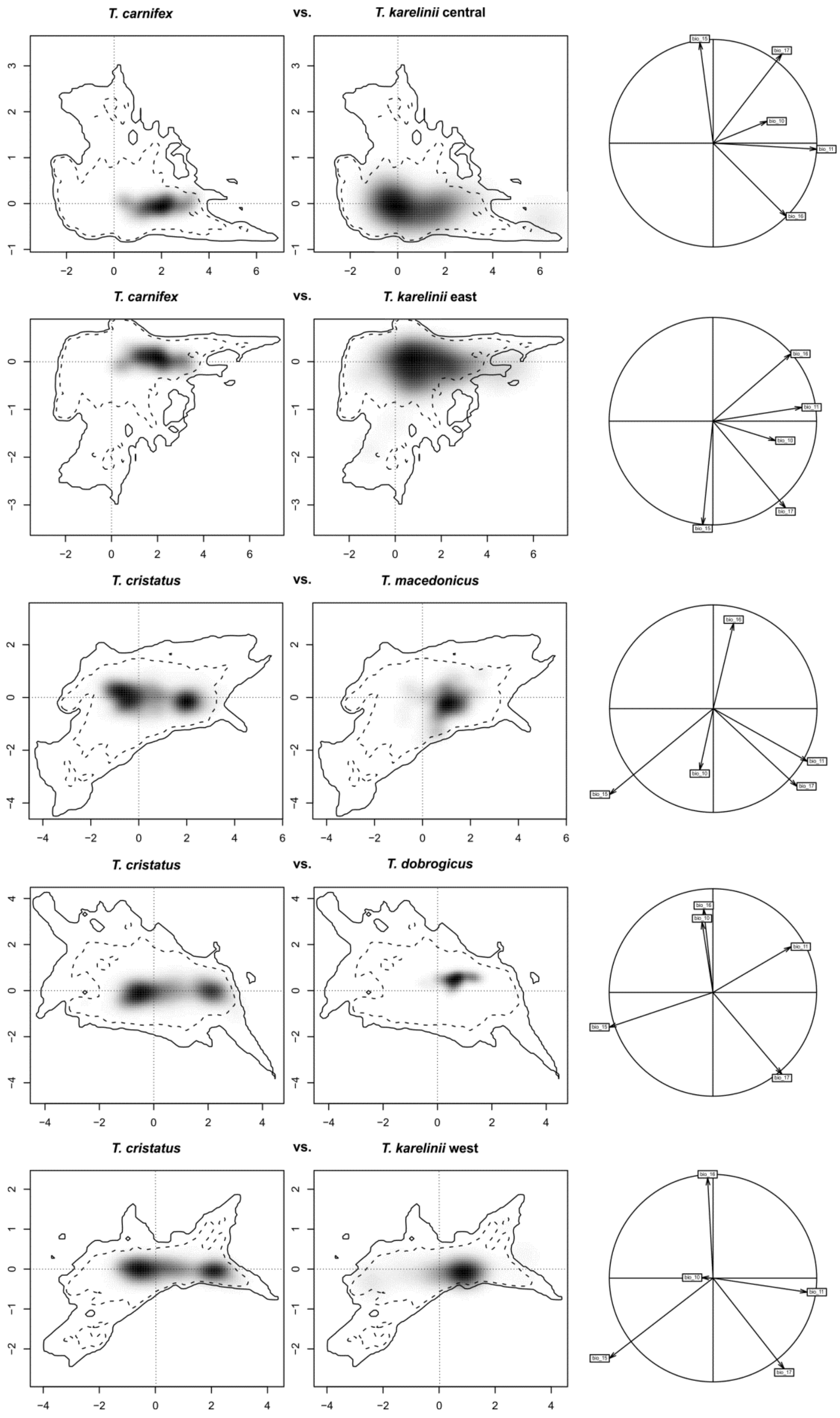
T. carnifex
vs.
T. karelinii central
T. carnifex
vs.
T. karelinii east
T. cristatus
vs.
T. macedonicus
T. cristatus
vs.
T. dobrogicus
T. cristatus
vs.
T. karelinii west

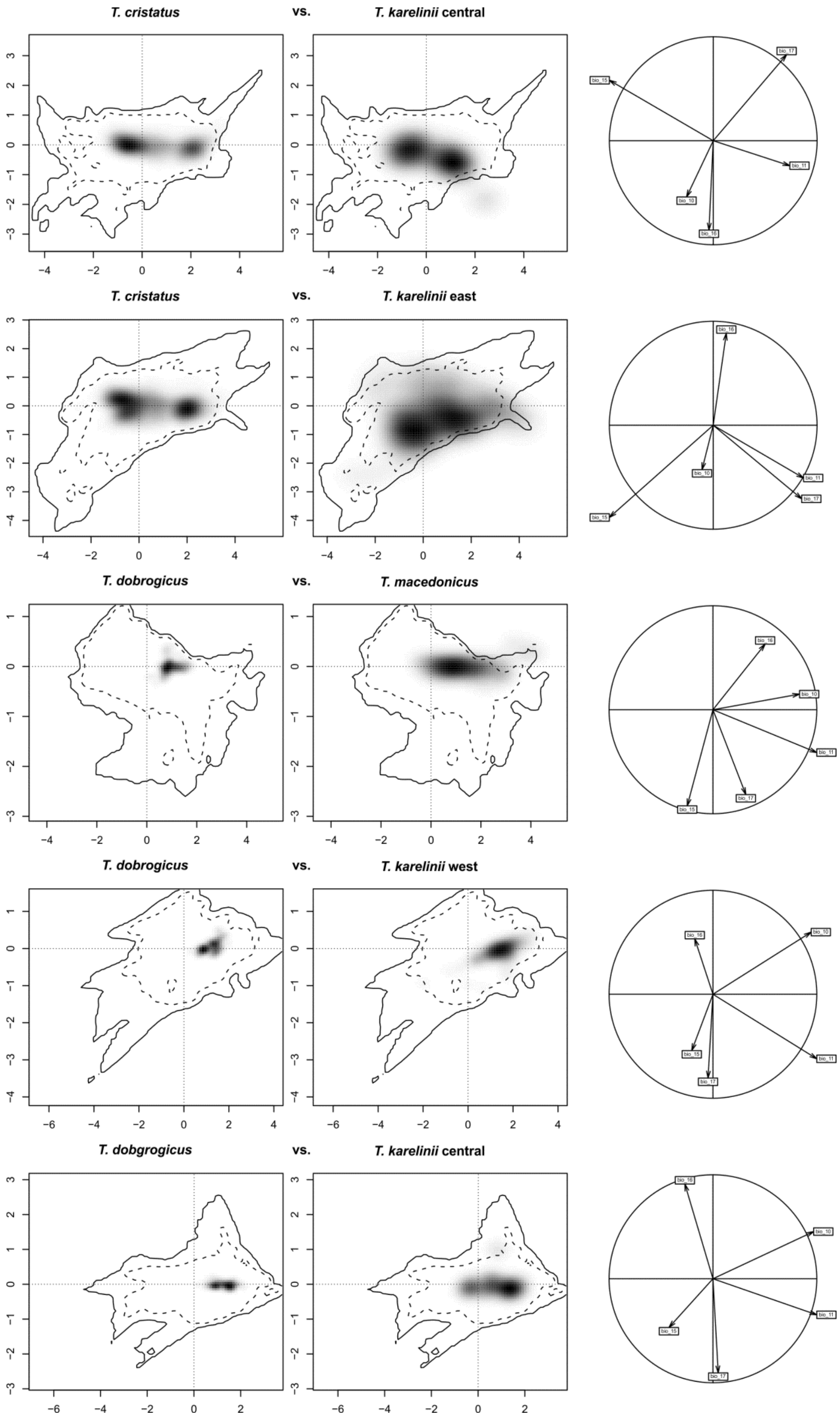
T. cristatus
vs.
T. karelinii central
bio_17
bio_15
bio_11
bio_10
bio_16
T. cristatus
vs.
T. karelinii east
bio_16
bio_10
bio_11
bio_17
bio_15
T. dobrogicus
vs.
T. macedonicus
bio_16
bio_10
bio_11
bio_17
bio_15
T. dobrogicus
vs.
T. karelinii west
bio_16
bio_10
bio_15
bio_17
bio_11
T. dobgrogicus
vs.
T. karelinii central
bio_16
bio_10
bio_11
bio_15
bio_17

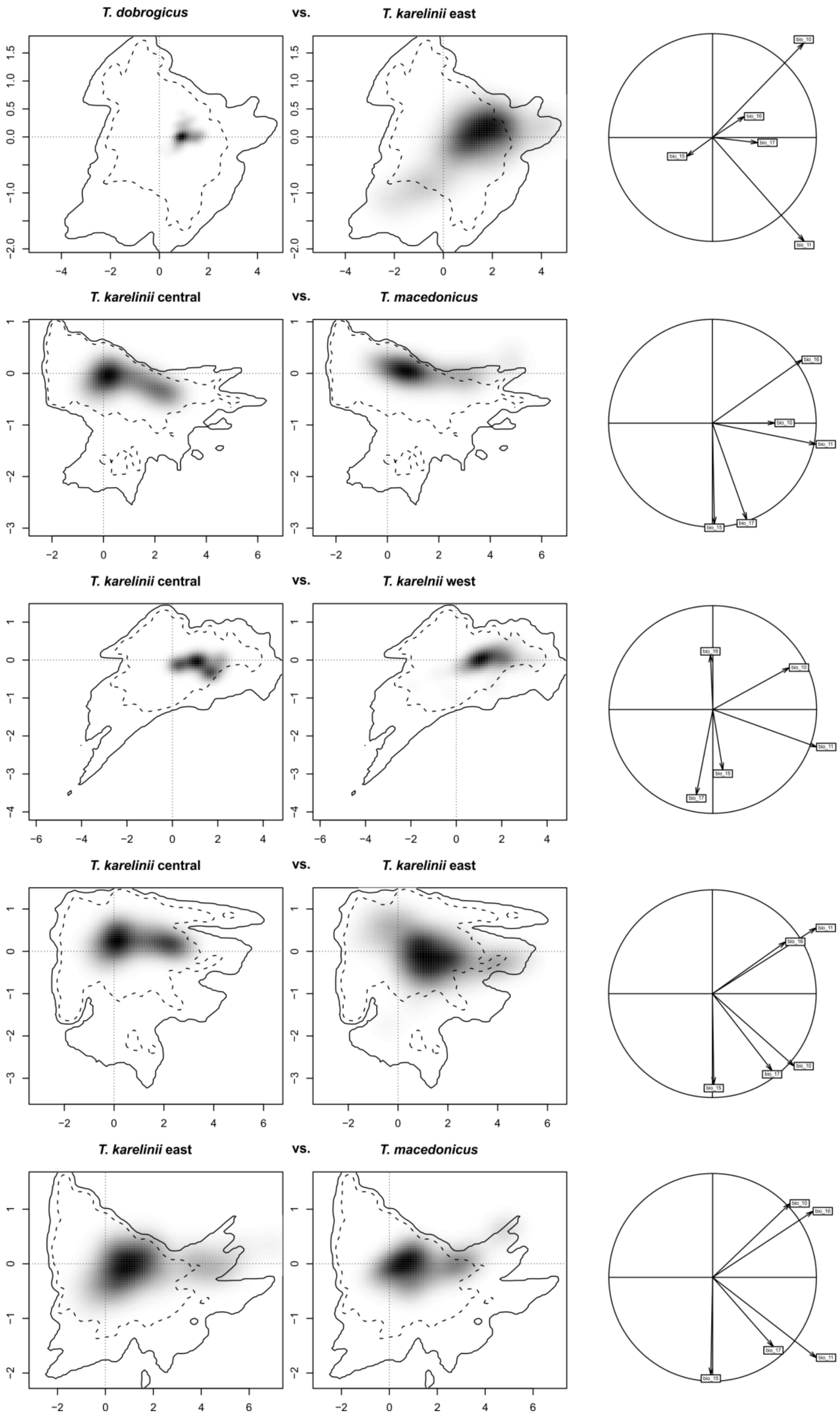
T. dobrogicus
vs.
T. karelinii east
bio_10
bio_16
bio_17
bio_15
bio_11
T. karelinii central
vs.
T. macedonicus
bio_16
bio_10
bio_11
bio_15
bio_17
T. karelinii central
vs.
T. karelnii west
bio_16
bio_10
bio_11
bio_15
bio_17
T. karelinii central
vs.
T. karelinii east
bio_11
bio_16
bio_10
bio_17
bio_15
T. karelinii east
vs.
T. macedonicus
bio_10
bio_16
bio_17
bio_11
bio_15

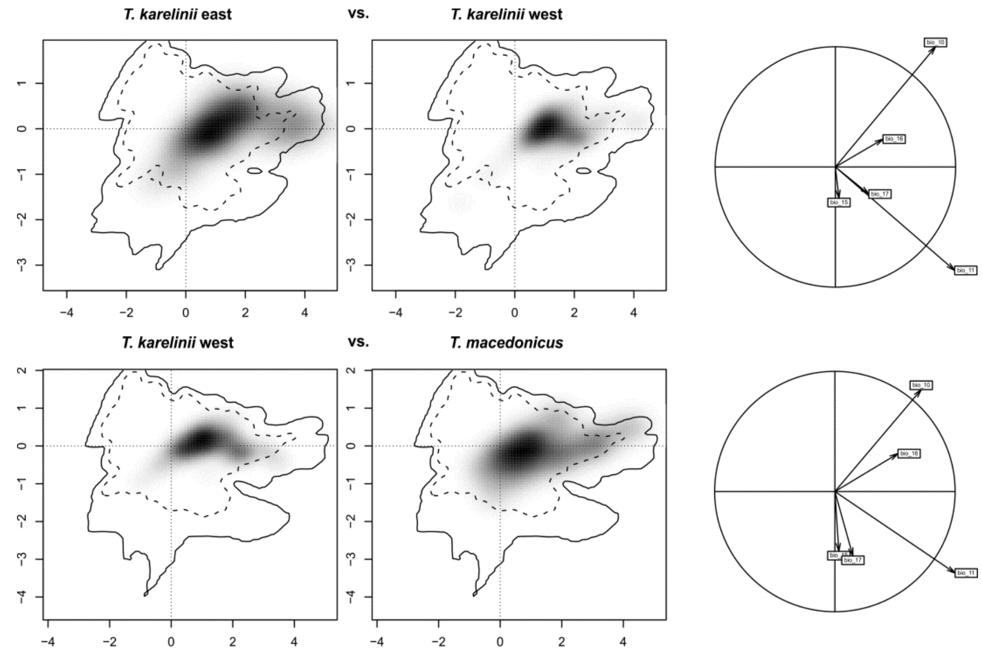

T. karelinii east
vs.
T. karelinii west
T. karelinii west
vs.
T. macedonicus
bio_10
bio_16
bio_17
bio_15
bio_11
bio_10
bio_16
bio_17
bio_11
